# The Prediction of a New CLCuD Epidemic in the Old World

**DOI:** 10.3389/fmicb.2017.00631

**Published:** 2017-04-19

**Authors:** Muhammad N. Sattar, Zafar Iqbal, Muhammad N. Tahir, Sami Ullah

**Affiliations:** ^1^Department of Environment and Natural Resources, Faculty of Agriculture and Food Science, King Faisal UniversityAl-Hasa, Saudi Arabia; ^2^Akhuwat-Faisalabad Institute of Research, Science and TechnologyFaisalabad, Pakistan; ^3^Independent Researcher, BeltsvilleMD, USA; ^4^University College of Agriculture, University of SargodhaSargodha, Pakistan

**Keywords:** CLCuD, monopartite begomovirus, bipartite begomovirus, Multan epidemic, Burewala epidemic, SNPs

## Abstract

Cotton leaf curl disease (CLCuD), the most complex disease of cotton, is a major limiting biotic factor to worldwide cotton productivity. Several whitefly-transmitted monopartite begomoviruses causing CLCuD have been characterized and designated as CLCuD-associated begomoviruses. Despite of being reported over 100 years ago in Africa, CLCuD became economically pandemic causing massive losses to cotton production in Pakistan and India during past couple of decades. In Asia, cotton has faced two major epidemics during this period viz. “Multan epidemic” and “Burewala epidemic.” The “Multan epidemic” era was 1988–1999 after which the virus remained calm until 2002 when “Burewala epidemic” broke into the cotton fields in Indo-Pak subcontinent, till 2013–2014. However, both the epidemics were caused by monopartite begomovirus complex. Similarly in Africa, *Cotton leaf curl Gezira virus* with associated DNA-satellites causes CLCuD. Quite recently, in the Old World (both Asia and Africa), bipartite begomoviruses have started appearing in the areas under cotton cultivation. Under such aggravated circumstances, it seems we are heading toward another epidemic of CLCuD in the Old World. Here we articulate the causes and potential emergence of the third epidemic of CLCuD in Asia. The current situation of CLCuD in Asia and Africa is also discussed.

Cotton leaf curl disease (CLCuD) is a major threat to worldwide cotton productivity. Indo-Pak subcontinent is a paradigm where cotton production has been severely compromised in past few decades because of the onset of two CLCuD epidemics. India and Pakistan are third and fourth largest cotton producers in the world with 6.05 and 2.17 million ton annual production, respectively (FAOStat, 2013). The CLCuD, showing characteristic symptoms of upward or downward leaf curling, vein swelling, leaf enation, and growth stunting, is transmitted by a ubiquitous whitefly (*Bemisia tabaci*) and is caused by a complex of five single-stranded DNA viruses belonging to genus *Begomovirus* (family *Geminiviridae*) along with their DNA satellites, viz. betasatellite and alphasatellite (Briddon and Stanley, 2006; Zhou, 2013; Brown et al., 2015). Understanding the dynamics of this disease is essential in order to sustain the worldwide cotton productivity and socio-economic values of the people.

Two major CLCuD complexes are prevailing in the Old World, i.e., “the African complex” and “the Asian complex.” In Africa, CLCuD was reported, for the first time, in 1912 from Nigeria, later from Sudan in 1924 and Tanzania in 1926 (Farquharson, 1912; Kirkpatrick, 1931). In Africa, since 2002 a single begomovirus species *Cotton leaf curl Gezira virus* (CLCuGeV) associated with Cotton leaf curl Gezira betasatellite (CLCuGeB) and Cotton leaf curl Gezira alphasatellite (CLCuGeA) has been predominantly the causative agent of CLCuD (Idris and Brown, 2002; Idris et al., 2005). However, very recently a bipartite begomovirus *Cotton yellow mosaic virus* (CYMV) has been reported for the first time in cultivated cotton (*Gossypium raimondii*) from the west and central Africa (Leke et al., 2016). Unlike Africa, mapping the exact history of CLCuD is difficult in Asia, however, in Pakistan introgression of high yielding but highly susceptible varieties of cotton (*Gossypium hirsutum*) favored the onset of this disease (Ali, 1997). During 1967 leaf curl symptoms, as a minor sporadic nuisance, were observed in a small cotton field near Multan, Pakistan but remained un-noticed (Hussain and Ali, 1975); this was the first inception of CLCuD. The virus [most probably *Cotton leaf curl Multan virus* (CLCuMuV)] kept on proliferating and burst into an epidemic in 1988 at Moza Kokhran, Multan, Pakistan. The country-wide spread of CLCuD most likely started from this vicinity and later moved across the borders into the northwestern states of India. In India, the first evidence of CLCuD was reported near border area Sri Ganganagar in 1989, which later spread as an epidemic in 1994 (Monga et al., 2004). During 1990s, up to 80% yield losses were recorded in Pakistan that caused a shortfall of about US $5 billion to Pakistan’s economy (Briddon and Markham, 2000). Later in 1998, three different species of begomoviruses were found to be associated with the CLCuD-first epidemic (**Figure 1A**), which today are named: *Cotton leaf curl Alabad virus* (CLCuAlV), *Cotton leaf curl Kokhran virus* (CLCuKoV), and CLCuMuV (Zhou et al., 1998). This epidemic has been known as the “Multan epidemic” (Sattar et al., 2013). Later, other begomoviruses such as *Tomato leaf curl Bangalore virus* (ToLCBaV) and *Papaya leaf curl virus* (PaLCuV) were also identified infecting cotton (Zhou et al., 1998; Mansoor et al., 2003; Kirthi et al., 2004). Interestingly, all these three CLCuD-associated begomoviruses (CABs) were found associated with a single species of betasatellite Cotton leaf curl Multan betasatellite (CLCuMuB) and satellite-like component Cotton leaf curl Multan alphasatellite (CLCuMuA) (**Figure 1A** and Supplementary Table S1) (Mansoor et al., 1999). The major reason for Multan epidemic is unanimously considered to be the cultivation of highly susceptible *G. hirsutum* cultivar S-12, which was cultivated on 46% area in Punjab, Pakistan (Briddon and Markham, 2000).

**FIGURE 1:**
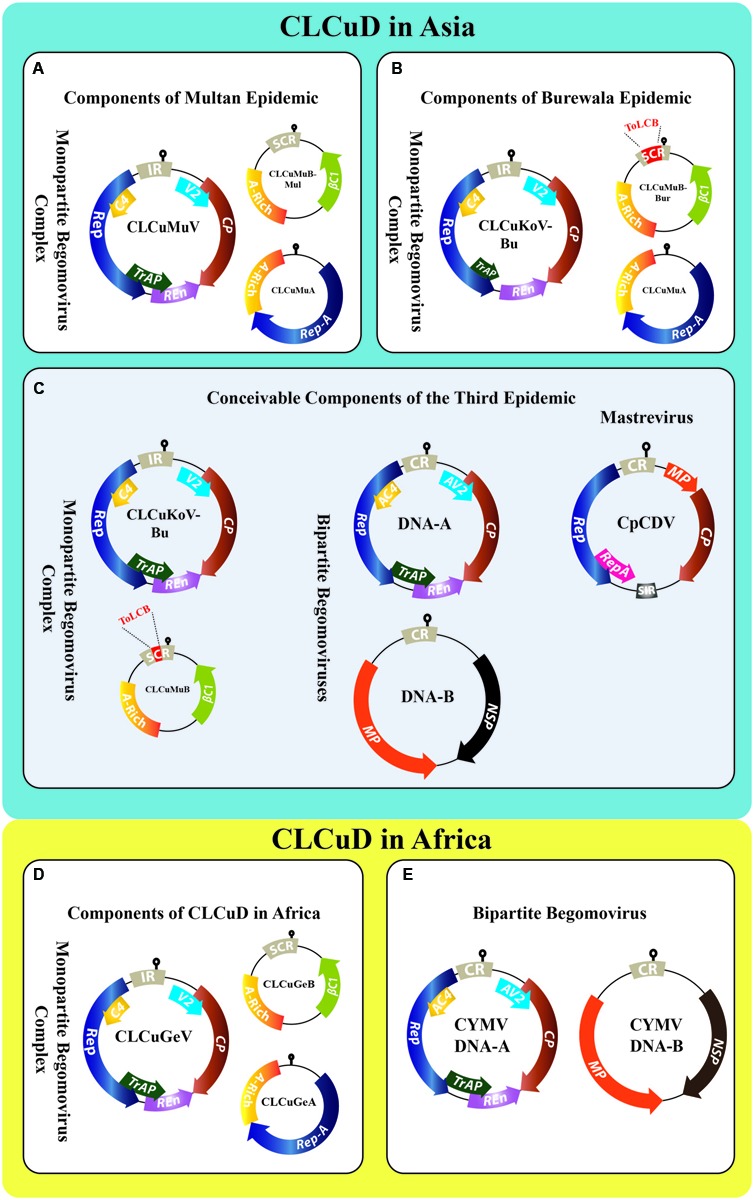
**Genome organization of all the contributing viruses of CLCuD in Asia (A–C)** and Africa **(D,E)**. The key begomovirus along with DNA satellites of the “Multan epidemic” **(A)**, “Burewala epidemic” **(B)**, and of the proposed “third epidemic” **(C)** are shown. During the Multan epidemic era, several begomoviruses were reported concomitantly from cotton plants, i.e., CLCuAlV, CLCuKoV, PaLCuV, and ToLCBaV. However, the key begomovirus was CLCuMuV. The Burewala epidemic involved a truncated transcriptional activator protein (TrAP; short green arrow) and a recombinant CLCuMuB^Bur^ having a stretch of ∼98 nt in the SCR region (highlighted in red) acquired from ToLCB **(B)**. The third epidemic in Asia may either involve CLCuKoV-Bu having intacted TrAP along with CLCuMuB having a stretch of ∼24 nt from ToLCB (highlighted in red); or ToLCNDV (DNA-A and DNA-B), ToLCV (DNA-A) together with ToLCNDV (DNA-B); or a *Mastrevirus* CpCDV **(C)**. In parallel, the CLCuD situation in Africa involved monopartite begomovirus complex **(D)** and recently bipartite begomovirus CYMV (DNA-A and DNA-B) **(E)**. The genomes of monopartite begomoviruses in the CLCuD-complex have single component DNA-A, which encodes two open reading frames (ORFs), i.e., V2 protein and coat protein (CP) in the virion-sense orientation whereas, four ORFs in the complementary-sense orientation are: replication associated protein (Rep), TrAP, replication enhancer protein (REn), and C4 protein (undermined functions). The bipartite begomovirus DNA-A resembles monopartite genome, however, they own a second genomic component DNA-B encoding two ORFs, i.e., nuclear shuttle protein (NSP) and movement protein (MP) in the virion- and complementary-sense orientation. The monopartite begomoviruses are also associated with DNA-satellites called alpha- and betasatellite. The alphasatellite encodes a single ORF (Rep-A) for its autonomous replication. Betasatellite encodes its single ORF βC1 in the complementary-sense orientation, which facilitates the helper begomovirus for its diverse functions.

In the late 1990s, after the Multan epidemic era (1989–1999), the cotton productivity was reinstated by developing resistant cultivars in Pakistan. However, theatrical appearance of a natural recombinant species that lacks a *C2* gene (**Figure 1B**), named Cotton leaf curl Burewala virus [now known as CLCuKoV-Burewala strain (CLCuKoV-Bu)] in the year 2002–2003, broke this resistance and all the resistant germplasm became susceptible to this unique member of CLCuD (Amrao et al., 2010b). This was the start of the second epidemic of CLCuD in this region and here referred as “Burewala epidemic” (Sattar et al., 2013). It was speculated that dispense of *C2* gene in CLCuKoV-Bu was a key player in resistance breaking (Amrao et al., 2010b; Briddon et al., 2014). However, the recent reports contradict this speculation as new field isolates of CLCuKoV-Bu with intact *C2* gene have been reported from India (Kumar et al., 2015; Godara et al., 2016). Another interesting twist in this story was the appearance of “Burewala strain” of CLCuMuB (CLCuMuB^Bur^), which was a recombinant of CLCuMuB and Tomato leaf curl betasatellite (ToLCB; Amin et al., 2006) (**Figure 1B**). The significance of both these invaders was the breakdown of established resistance in all the CLCuD-resistant cotton germplasm. The reasons for the ultimate selection of these recombinants through the use of resistant cultivars are not yet fully understood. According to Amin et al. (2006), the recombinant stretch of CLCuMuB^Bur^ may enhance the interaction between the betasatellite and the helper begomovirus.

Until this period, the southern parts of Pakistan were disease free but following the resistance breakdown, CLCuKoV-Bu was extended to Sindh province as well as to northwestern India in 2005 (Rajagopalan et al., 2012). Moreover, CLCuGeV—one of the CABs causing CLCuD in Africa—trans-replicating CLCuMuB and Chili leaf curl betasatellite has been also found from cotton in Pakistan (Tahir et al., 2011). In these areas the CLCuD is characterized by CLCuKoV, CLCuKoV-Bu, CLCuKoV-Shahdadpur strain (CLCuKoV-Sha; old name Cotton leaf curl Shahdadpur virus), and CLCuGeV along with two strains of CLCuMuB, i.e., “Burewala strain” and “Shahdadpur strain” (Amrao et al., 2010a). The factors driving the rapid evolution of these new strains of CLCuMuB in this region are not yet clear (Akhtar et al., 2014).

Until the advent of Burewala epidemic, which remained active from 2002 until 2013–2014, only monopartite begomoviruses associated with DNA-satellites were found from the infected cotton plants. But quite recently, like in Africa, bipartite begomoviruses are also reportedly appearing in cotton plants including *Tomato leaf curl virus* (ToLCV) (Zaidi et al., 2015) and *Tomato leaf curl New Delhi virus* (ToLCNDV) (Zaidi et al., 2016) (Supplementary Table S1), in Pakistan. Moreover, the identification of *Okra enation leaf curl virus* (OELCuV) (Hameed et al., 2014) and a *Mastrevirus Chickpea chlorotic dwarf virus* (CpCDV) (Manzoor et al., 2014) has further aggravated the scenario. It has already been proven that ToLCNDV synergistically interacts with CLCuKoV-Bu thereby enhancing the accumulation of CLCuMuB^Bur^ (a member of the Burewala epidemic of CLCuD) in the infected plants (Zaidi et al., 2016). The DNA-A of ToLCNDV has been shown empirically to trans-replicate CLCuMuB in the absence of DNA-B (Saeed et al., 2007) and also can cause CLCuD symptoms in cotton (Saeed, 2010). During a most recent study, ToLCNDV has been found associated with CLCuD-infected cotton over a vast area in Punjab and Sindh provinces of Pakistan (Zaidi et al., 2016). ToLCNDV has been prevailing in Indo-Pak subcontinent from nearly three decades (Padidam et al., 1995). It has not only been established in Asia (Mizutani et al., 2011; Yazdani-Khameneh et al., 2016) but also spread to other continents including Africa (Mnari-Hattab et al., 2015) and Europe to Spain (Juarez et al., 2014; Ruiz et al., 2015) and Italy (Panno et al., 2016). If we have learnt some lesson from the previous epidemics, a wise interpretation could be that this is not just an accidental ephemeral intrusion of a bipartite begomovirus in cotton. Rather, it may be an alarm bell for the third epidemic of CLCuD in this area (**Figure 1C**). The ability of ToLCNDV to develop synergistic interactions with other begomoviruses has recently been shown to cause resistance breakdown in chilies (Singh et al., 2016). CLCuAlV has been found associated with and trans-replicating a recombinant DNA-B of a bipartite begomovirus in wild cotton germplasm, proving the ability of CABs to acquire components from bipartite begomoviruses (Nawaz-ul-Rehman et al., 2012). Thus, if we foresee correctly, the third epidemic might involve CLCuKoV-Bu (with intact C2) associated with CLCuMuB and in synergism with ToLCNDV in the Indo-Pak subcontinent. While in Sindh province, ToLCNDV, CLCuKoV-Bu, and CLCuGeV along with local DNA-satellites may represent the key players of the third epidemic.

The Asian and African CLCuD complexes are quite similar in having monopartite begomoviruses associated with DNA-satellites as causative agents (**Figure 1**). Yet this similarity has been extended to the possibility of another epidemic in both continents because of the identification of bipartite begomovirus from the field cotton in Africa (Leke et al., 2016) and Asia (Zaidi et al., 2015, 2016) (**Figures 1C–E** and Supplementary Table S1). The monopartite begomoviruses infection in cotton is the main cause of yield losses in Asia and Africa; however, the appearance of bipartite begomoviruses in the cotton crop fields is a stark reminder of another destructive epidemic. It seems that both the continents are going through a paradigm shift of CLCuD complex. We may speculate that a change in vector population, host plant–virus interactions, injudicious use of insecticides and other environmental factors could be the key players that are exacerbating the occurrence of CLCuD. Environmental changes concomitantly affect plant–virus–vector interactions and members of this complex consequently try to confront such fluctuations through differential expression of genes, mutation, and recombination. In such three-way interaction, virus induces multiple dramatic physiological changes, either in vector and/or host, to increase the transmission. This scenario has another level of complexity associated with particular species of whitefly. Studies show that higher incidence of CLCuD in Punjab (Pakistan) and northwestern India is associated with Asia II-1 species of *B. tabaci*, the single predominant whitefly specie in these regions (Ahmad et al., 2010; Hameed et al., 2012; Rana et al., 2012) which plays an important role in the transmission of CLCuD. The other factors include the increased, unwise and injudicious use of insecticides and higher adaption rate of *Bacillus thuringiensis* (BT)-cotton hybrids in Indo-Pak subcontinent. The cultivation of BT-crops not only affects the pest populations in an area but could also alter the weed flora in a particular region (Albajes et al., 2011) and thus indirectly may change the virus population in a particular niche. Among other problems, the higher incidence of CLCuD on unapproved BT varieties has become a major issue both in India and Pakistan. The rate of CABs infection was 54.24% in 2008, which increased to 83.1% in 2009 after BT-cotton cultivation (Kakakhel, 2009) in Pakistan. Similarly, the extensive cultivation of BT-cotton hybrids in north Indian states of Haryana, Punjab, and Rajasthan led to increase virus inoculum in the Indian ecosystem (Kranthi, 2014). The high rate of BT-cultivation in Indo-Pak subcontinent might have played an indirect role in proliferation of virus inoculum in this region because most of the BT varieties have higher susceptibility to CABs as compared to non-BT (Ali et al., 2010; Kranthi, 2014, 2015). Effects of BT crops on the insect vectors (and indirectly promoting virus proliferation) have been reported for *Maize rough dwarf virus* (MRDV) and Fijiviruses from maize in Spain (Pons et al., 2005; Grilli and Bruno, 2007; Achon and Alonso-Dueñas, 2009). Another alarming nuance is the hyper mutation rate of CABs (∼2.706 to 4.96 × 10^-4^), which is comparable to many RNA viruses (Saleem et al., 2016). It is thus conceivable that high mutation rate and environmental paradigm shift can lead to rapid evolution and emergence of more virulent species and/or strains of CABs, leading to a situation that we fear today, the “third epidemic” of CLCuD in Indo-Pak subcontinent.

The correlation between various environmental factors and the vector-borne disease incidence has been a mechanistic determinant to assess the geographic spread of the disease risk (Chabot-Couture et al., 2014). Such correlations may also help beforehand disease assessments and to build models for their vector population (Grover-Kopec et al., 2005; White et al., 2011). A successful prevention of an emerging disease epidemic depends upon the early detection and timely control strategies. A number of plant disease epidemics are exemplary due to failure in timely detection and management, such as chestnut blight (Freinkel, 1997), white pine blister rust (Maloy, 1997) and citrus canker (Gottwald, 2007), etc. To validate our assumptions that “why this area (especially Multan city) has become such a ‘hot spot’ for new intrusions in cotton?” and to decipher the relation between CLCuD incidence and different meteorological parameters, “R-statistical and computing tool” was employed (R Development Core Team, 2016). A positive and highly significant correlation (0.85^∗∗∗^) between the disease severity and the disease incidence with respect to humidity and temperature was observed in all cotton growing areas of Pakistan (**Figure 2A**). Moreover, positive correlations between and among disease incidence, humidity (0.15), and temperature (0.25) support our hypothesis. Concomitant to this, disease incidence data of 19 years (1997–2015) along with the “Burewala epidemic” were mapped on a bar graph. Likewise to the Burewala epidemic during the years 1999–2002 when the disease incidence was dramatically at its lowest incidence (**Figure 2B**), similar disease scenario is prevailing in cotton growing areas of Pakistan (as shown in circle; **Figure 2B**) (2014–2015). Usually, during long hibernation/incubation periods, some pathogens remain cryptic while their infection continuously spreading (Tomlinson and Potter, 2010). This stage is pointing toward the likelihood of another epidemic of CLCuD in the cotton fields, which is supporting our hypothesis. Rapidly emerging begomoviruses bear huge numbers of single nucleotide polymorphism (SNP) in their genome to evolve and/or evade the host defense response. To assess the SNPs diversity in CABs, year-wise virus sequences were retrieved from the NCBI database, aligned by keeping the oldest known viral sequence as a master sequence and then SNPs were calculated semi-manually (NCBI-multiple sequence alignment tools) and by online web-based tool^1^. The SNPs with ≥75% frequency were mapped on the genomic structure of the respective virus (**Figure 3**). The present study demonstrates that an extensive SNPs diversity is associated with CABs genome and a highest SNPs diversity exists in CpCDV followed by ToLCNDV, CLCuGeV, and CLCuMuV, respectively, while least SNPs were evident in CLCuKoV-Bu genome (**Figure 3**). The occurrence of fewer SNPs in CLCuKoV-Bu may likely be due to recombination event from which this virus has emerged out. Further analysis revealed that >30% of the SNPs were non-synonymous, thus can have substantial role in the virus divergence and evolution.

**FIGURE 2:**
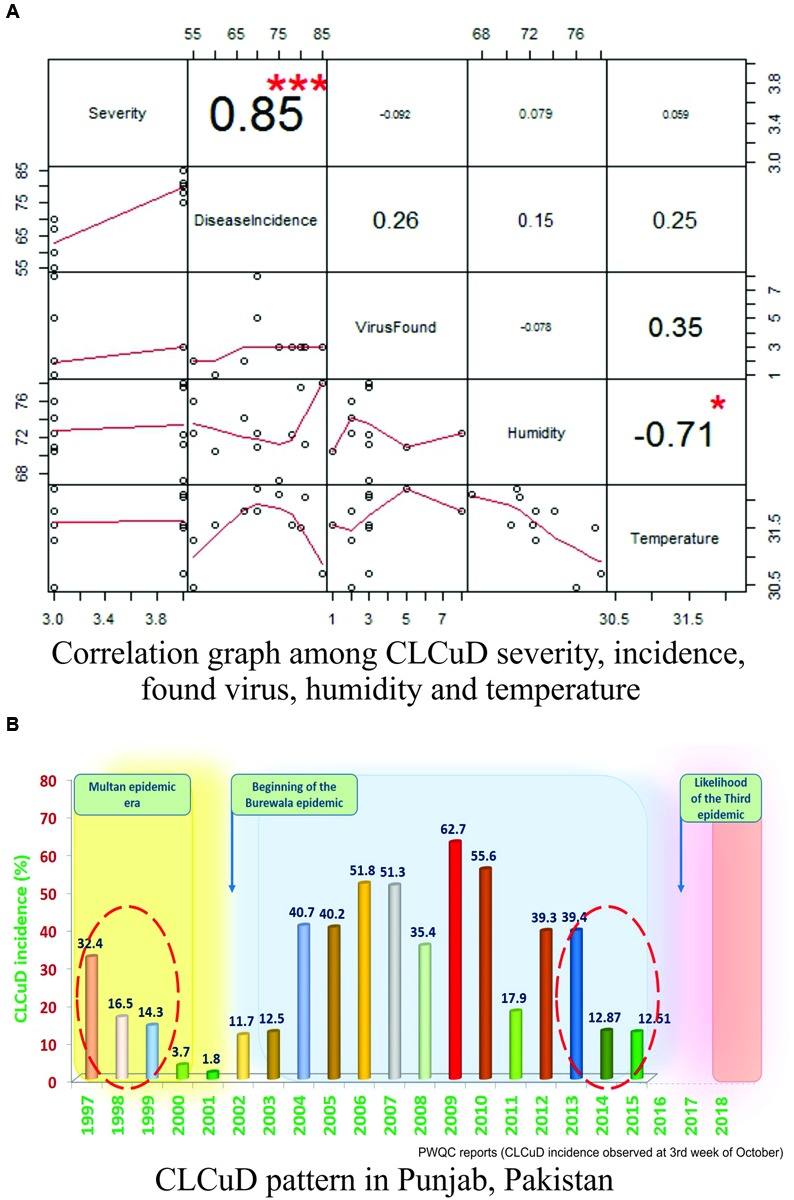
**Statistical modeling of CLCuD in Pakistan. (A)** The R-Plot represents the correlation structure in between disease severity, disease incidence, virus found, humidity, and temperature. Positive and significant correlations were calculated as numeric values among all the parameters. The numeric values with larger font and positive numbers denote higher correlation strength and vice versa. The significance of each correlation was represented by asterisks, i.e., “^∗∗∗^” represents very high significance and “^∗^” represents less significance (*P*-values <0.01), respectively. All the correlations were also represented in graphical forms (as line segments in red color) and their respective scales are given along *x*- and *y*-axis. **(B)** Graphical bar chart, representing 19 years data (1997–2015) of CLCuD incidence pattern in the Punjab province, Pakistan. The time scale of CLCuD was plotted against the CLCuD incidence (%). Each bar having numeric value and different color (green for least and red for highest incidence) represents the CLCuD incidence (%) in the respective year. The yellow shaded area represents the Multan epidemic era until the year 1999. Whereas, the gray shaded area represents the Burewala epidemic era starting from the year 2002 until 2013–2014. From here on, likely a new epidemic is being evolved (shaded area in pink color). The shaded area in between the epidemics represents the hibernation period of the virus(es). The red dotted circles are highlighting CLCuD incidence scenarios prior to the onset of a new disease outbreak.

**FIGURE 3:**
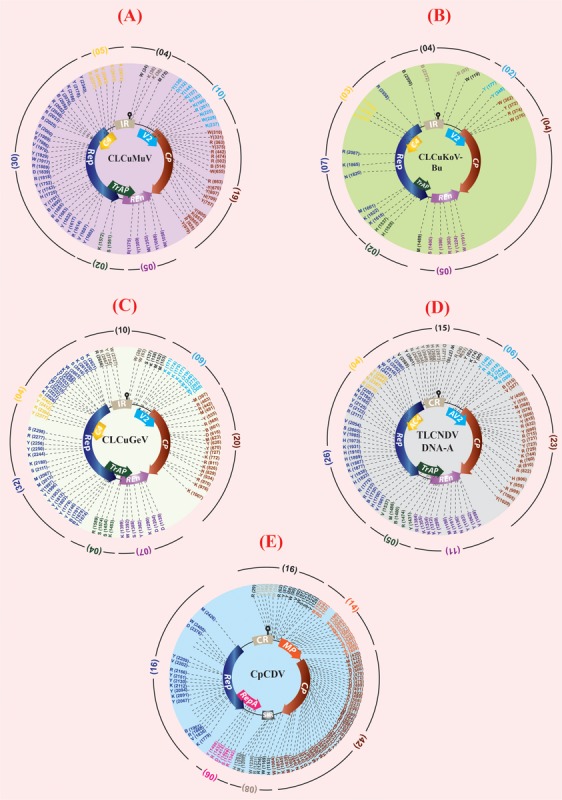
**Graphical representation of the location and type(s) of single nucleotide polymorphisms (SNPs) in CLCuD-associated begomoviruses (CABs) DNA-A (A–D)** and *Mastrevirus* **(E)**. Maximum available genome sequences (∼40–100 for each candidate virus) were retrieved from NCBI GenBank database. Semi-manual (NCBI based multiple sequence alignment tools) and web-based tools were used for SNPs identification and classification following the International Union of Pure and Applied Chemistry (IUPAC). For CLCuMuV, CLCuKoV-Bu, and CLCuGeV the oldest reported virus sequence was set as reference sequence for alignment and to locate the viral SNPs. Whereas, in case of ToLCNDV and CpCDV, the sequences reported from cotton crop were set as reference sequence. In the second method, a web-based tool (www.fludb.org/brc/snpAnalysis) was used and all the aligned sequences of the CABs were subjected to SNPs calculation. Only those nucleotide positions were marked as SNPs, which had a quality score ≥40% in both the methods used. The SNPs in the non-coding regions are shown in black and gray text whereas, the SNPs in the coding regions are shown in the respective colors of their ORFs. The total number of SNPs found in the ORFs of each molecule is mentioned separately along-sides (in brackets), respectively.

Thus, we may speculate that today we come across a similar situation of early 2000s in terms of virus incubation period (**Figure 2B**). Based upon our statistical modeling and the currently available data, we may infer that the Multan, Pakistan (the place for Multan- and Burewala-epidemic origin) may face a serious eruption of CLCuD with new arsenals, which may spread across the borders.

Various strategies encompassing classical breeding and biotechnological techniques have been employed to control the CLCuD (Iqbal et al., 2016). All these techniques showed a laconic control over CLCuD. In a three-way interaction of cotton–whitefly–CABs (along with DNA-satellites), none (either breeding or biotechnological techniques) have ever been tried to target this complex simultaneously. Recently, a CRISPR/Cas9-based multiplexing technique has been proposed (Iqbal et al., 2016) as an ultimate strategy to target whole CLCuD complex, i.e., the CABs as well as associated DNA-satellites. However, authors hereby would like to propose that the third epidemic could be obstructed by targeting more than one component of the cotton–whitefly–CABs complex through multiplexing CRISPR/Cas9 system.

## Concluding Remarks

Whitefly populations responsible for transmitting CLCuD appear to be favored by the humid and high temperature conditions in the Indo-Pak subcontinent, where cotton is a key crop. Due to expansion in the geographic range of CABs from South Asia to China, a dynamic change in CABs in South Asia is an emerging threat to cotton production (Cai et al., 2010). The key factor is whitefly being a vector for both monopartite and bipartite begomoviruses. The anticipated global warming, poor social practices, unwise use of insecticides, extensive cultivation of unapproved CLCuD susceptible BT-cotton hybrids and non-BT germplasm, mono-cropping system, transportation of infected plants (especially ornamental plants) and higher mutation and recombination rate in the virus genome will exacerbate the whitefly-transmitted CABs. Ultimately, it may enhance the likelihood of another epidemic to occur due to the emergence of more virulent species/strains. It is therefore of a dire need to devise more effective and sustainable approaches to achieve control over host plant–virus–vector complexes. The best way to control CLCuD is the exploitation of natural resistance by stacking multiple resistance sources together, i.e., through modern genetic tools. Understanding of various aspects of pathosystem like host–vector interaction, insect vector alternative hosts, biological role of new begomoviruses involvement in the CLCuD and environmental factors will also help to manage unpleasant surprises in the future.

## Author Contributions

MS conceived the idea, created the figures and drafted the first draft. ZI wrote part of the manuscript, collected meteorological and CLCuD incidence data, and helped in preparation of the first draft. MT helped in retrieving all the data and prepared the tables. SU performed all statistical analysis. Final draft was checked and approved by all authors.

## Conflict of Interest Statement

The authors declare that the research was conducted in the absence of any commercial or financial relationships that could be construed as a potential conflict of interest.

## Footnotes

1 www.fludb.org/brc/snpAnalysis
