# Supplementary material for: The Prediction of a New CLCuD Epidemic in the Old World
Source: Front Microbiol. 2017 Apr 19;8:631. doi: 10.3389/fmicb.2017.00631 (PMC5395620; doi:10.3389/fmicb.2017.00631)
Supplement: Supplementary file 1 [file Table_1.docx]

**-- Frontiers in Microbiology --**

**A Perspective Overview**

**The prediction of a new CLCuD epidemic in the Old World**

Muhammad Naeem Sattar^1*†^, Zafar Iqbal^2†^, Muhammad Nouman Tahir^3^, Sami Ullah^4^

^1^Department of Environment and Natural Resources, Faculty of Agriculture and Food Science, King Faisal University, PO Box 420, Al-Hasa 31982, Saudi Arabia.

^2^Akhuwat-FIRST, University Park, Faisalabad, Pakistan.

^3^Maryland, USA.

^4^ University college of Agriculture, University of Sargodha, Sargodha, Pakistan

**Running Title:** Prospects of another CLCuD epidemic

^*^Correspondence:

Muhammad Naeem Sattar

E-mail: [Naeem.sattar1177@gmail.com](mailto:Naeem.sattar1177@gmail.com)

Phone: +966-582 123443

^†^These authors have contributed equally to this work.

**Key words:** CLCuD, monopartite begomovirus, bipartite begomovirus, Multan epidemic, Burewala epidemic, SNPs.

**Supplementary Table 1**: All the reported cotton leaf curl disease associated begomoviruses (CABs) and DNA-satellites along with their origin and accession numbers.

| **CLCuD-associated monopartite begomoviruses** | **Country** | **Year of Collection / Submission** | **Host** | **Accession number** |
| --- | --- | --- | --- | --- |
| ***Cotton leaf curl Multan virus* (CLCuMuV)** | | | | |
| **CLCuMuV - Darwinii** | Pakistan | 2006 | *Gossypium darwinii* | EU365613 |
|  |  | 2006 | *G. hirsutum* | FJ218487 |
|  | India | 2010 | *G. hirsutum* | JN678803 |
| **CLCuMuV - Faisalabad** | Pakistan | 1995 | *Abelmoschus esculentus* | AJ002447 |
|  |  | 1995 | *A. esculentus* | AJ002458 |
|  |  | 1992 | *G. hirsutum* | AJ496287 |
|  |  | 1992 | *G. hirsutum* | AJ496461 |
|  |  | 1995 | *G. hirsutum* | X98995 |
|  |  | 2006 | *G. hirsutum* subsp. *latifolium* | EU384573 |
|  | India | 2010 | *G. hirsutum* | JN678804 |
|  |  | 2010 | *G. hirsutum* | JN678806 |
|  |  | 2013 | *Hibiscus rosa-sinensis* | KR135370 |
|  | China | 2006 | *H. rosa-sinensis* | EF465535 |
|  |  | 2007 | *H. rosa-sinensis* | JN968573 |
|  |  | 2008 | *H. rosa-sinensis* | GQ503175 |
|  |  | 2009 | *H. rosa-sinensis* | HQ455355 |
|  |  | 2008 | *H. rosa-sinensis* | HQ455367 |
|  |  | 2008 | *A. esculentus* | FJ770370 |
|  |  | 2009 | *A. esculentus* | GU574208 |
|  |  | 2010 | *G. hirsutum* | GQ924756 |
|  |  | 2011 | *G. hirsutum* | JQ317603 |
|  |  | 2011 | *Malvaviscus penduliflorus* | JQ424826 |
|  |  | 2011 | *H. rosa-sinensis* | JQ943408 |
|  |  | 2011 | *H. rosa-sinensis* | JQ963625 |
|  |  | 2012 | *H. rosa-sinensis* | JX286656 |
|  |  | 2011 | *H. rosa-sinensis* | JX286662 |
|  |  | 2011 | *A. esculentus* | JX286664 |
|  |  | 2012 | *H. rosa-sinensis* | JX861210 |
|  |  | 2012 | *H. rosa-sinensis* | JX914662 |
|  |  | 2012 | *G. hirsutum* | KC171654 |
|  |  | 2012 | *H. cannabinus* | KF444948 |
|  |  | 2013 | *H. rosa-sinensis* | KF766955 |
|  |  | 2013 | *G. hirsutum* | KP762786 |
|  |  | 2015 | *A. manihot* | KU245932 |
|  | Philippines | 2012 | *H. rosa-sinensis* | KF413616 |
|  |  | 2012 | *H. rosa-sinensis* | KF413618 |
| **CLCuMuV- Hibiscus** | India | 2011 | *H. rosa-sinensis* | JN807763 |
|  |  | 2011 | *H. rosa-sinensis* | JN880418 |
| **CLCuMuV - Hisar** | Pakistan | 1997 | *G. hirsutum* | AJ132430 |
|  |  | 1996 | *A. esculentus* | AJ002459 |
|  |  | 2006 | *G. hirsutum* | FJ218486 |
|  | India | 1999 | *G. hirsutum* | AY765253 |
|  |  | 2005 | *G. hirsutum* | DQ191160 |
|  |  | 2010 | *Carica papaya* | JN558352 |
| **CLCuMuV - Pakistan** | Pakistan | 2006 | *G. davidsonii* | EU365616 |
|  |  | 2006 | *G. mustelinum* | EU384574 |
|  | India | 2005 | *G. hirsutum* | JF502359 |
| **CLCuMuV - Rajasthan** | India | 1994 | *G. hirsutum* | AF363011 |
|  |  | 1999 | *G. hirsutum* | AY765254 |
|  |  | 2003 | *G. hirsutum* | AY795605 |
|  |  | 2003 | *G. hirsutum* | JF509746 |
|  |  | 2005 | *G. hirsutum* | JF509750 |
|  |  | 2005 | *G. hirsutum* | DQ191160 |
|  |  | 2009 | *G. hirsutum* | HQ158009 |
|  |  | 2006 | *G. hirsutum* | HQ158011 |
|  |  | 2006 | *G. hirsutum* | HM235774 |
|  |  | 2007 | *G. hirsutum* | FN645912 |
|  |  | 2008 | *G. hirsutum* | GQ220850 |
|  |  | 2009 | *G. hirsutum* | HM037923 |
|  |  | 2010 | *G. hirsutum* | JF502361 |
|  |  | 2012 | *G. hirsutum* | KJ959628 |
|  |  | 2014 | *H. cannabinus* | KT390455 |
|  | Pakistan | 2005 | *Solanum lycopersicum* | AM501481 |
|  |  | 2008 | *Digera arvensis* | FM202328 |
| ***Cotton leaf curl Alabad virus* (CLCuAlV)** | | | | |
| **CLCuAlV - Alabad** | Pakistan | 1996 | *G. hirsutum* | AJ002452 |
|  |  | 1996 | *G. hirsutum* | AJ002455 |
| **CLCuAlV - Lobatum** | Pakistan | 2006 | *G. lobatum* | FJ210467 |
|  |  | 2006 | *G. lobatum* | FJ218488* |
| **CLCuAlV - Multan** | Pakistan | 2006 | *G. punctatum* | EU384575 |
|  |  | 2006 | *G. punctatum* | EU384578* |
|  |  | 2006 | *G. gossypioides* | FJ218485 |
| **CLCuAlV - Haryana** | India | 2005 | *A. esculentus* | GU112081 |
|  |  | 2004 | *G. hirsutum* | AY765257 |
|  |  | 2013 | *G. hirsutum* | KM096468 |
| **CLCuAlV - Karnal** | India | 2005 | *A. esculentus* | GU112004 |
| ***Cotton leaf curl Bangalore virus* (CLCuBaV)** | | | | |
| **CLCuBaV** | India | 2004 | *G. barbadense* | AY705380 |
|  |  | 2005 | *A. esculentus* | GU112003 |
| ***Cotton leaf curl Gezira virus* (CLCuGeV)** | | | | |
| **CLCuGeV - Sudan** | Sudan | 1996 | *G. hirsutum* | AF260241 |
|  |  | 2001 | *A. esculentus* | AY036006 |
|  |  | 2001 | Sida | AY036007 |
|  | Saudi Arabia | 2012 | *A. esculentus* | HG530540 |
| **CLCuGeV - Burkina Faso** | Burkina Faso | 2009 | *A. esculentus* | FN554540 |
|  | Niger | 2007 | *A. esculentus* | EU432374 |
|  |  | 2007 | *A. esculentus* | FJ469627 |
| **CLCuGeV - Cairo** | Egypt | 2003 | *Alcea rosea* | AJ542539 |
| **CLCuGeV - Hollyhock** | Egypt | 1997 | *A. rosea* | AF014881 |
|  |  | 1997 | *A. rosea* | AY036009 |
| **CLCuGeV - Cameroon** | Cameroon | 2008 | *A. esculentus* | FM210276 |
|  |  | 2007 | *A. esculentus* | HE793429 |
| **CLCuGeV - Egypt** | Egypt | 2000 | *A. rosea* | AF155064 |
|  |  | 2000 | *A. rosea* | AY036010 |
|  | Pakistan | 2005 | *G. hirsutum* | FR751142 |
|  | Jordan | 2009 | *A. rosea* | GU945265 |
|  | Israel | 2011 | Whiteflies feeding on squash | KT099132 |
|  | United Arab Emirates | 2013 | *A. esculentus* | KJ939446 |
| **CLCuGeV - Lysoka** | Cameroon | 2008 | *A. esculentus* | FM164726 |
|  |  | 2007 | *A. esculentus* | HE793426 |
| **CLCuGeV - Madagascar** | Madagascar | 2001 | *Phaseolus vulgaris* | AM701757 |
| **CLCuGeV - Mali** | Mali | 2006 | *A. esculentus* | EU024120 |
| **CLCuGeV - Niger** | Niger | 2007 | *A. esculentus* | EU432373 |
|  |  | 2007 | *A. esculentus* | FJ469626 |
|  | Burkina Faso | 2009 | *A. esculentus* | FN554541 |
|  | Ivory Coast | 2013 | *A. esculentus* | KX100570 |
| **CLCuGeV - Okra** | Sudan | 2007 | *A. esculentus* | FJ868828 |
| ***Cotton leaf curl Kokhran virus* (CLCuKoV)** | | | | |
| **CLCuKoV - Kokhran** | Pakistan | 1992 | *G. hirsutum* | AJ496286 |
|  |  | 1995 | *G. hirsutum* | AJ002448 |
|  |  | 1996 | *G. hirsutum* | AJ002449 |
|  |  | 2005 | *G. hirsutum* | FN552006 |
|  |  | 2008 | *G. stocksii* | HM468427 |
|  | India | 2003 | *G. hirsutum* | AY456683 |
|  |  | 2010 | *G. hirsutum* | HQ257374 |
| **CLCuKoV - Burewala** | Pakistan | 2006 | *G. hirsutum* | AM421522 |
|  |  | 2004 | *G. hirsutum* | AM774300 |
|  |  | 2005 | *Xanthium strumarium* | FR819707 |
|  |  | 2006 | *G. hirsutum* (octaploid) | EU365618 |
|  |  | 2006 | *G. hirsutum* (octaploid) | EU384572 |
|  |  | 2009 | *G. hirsutum* | FR750318 |
|  |  | 2009 | *H. rosa-sinensis* | HG003876 |
|  |  | 2010 | *G. hirsutum* | FR837932 |
|  |  | 2010 | *G. hirsutum* | HF549181 |
|  |  | 2011 | *G. hirsutum* | HF549184 |
|  |  | 2011 | *Luffa cylindrica* | HF567942 |
|  |  | 2012 | *Ricinus communis* | HE985227 |
|  | India | 2004 | *G. hirsutum* | JF502353 |
|  |  | 2009 | *G. hirsutum* | JF502357 |
|  |  | 2010 | *G. hirsutum* | JF502360 |
|  |  | 2010 | *G. hirsutum* | JF502373 |
|  |  | 2005 | *G. hirsutum* | JF509747 |
|  |  | 2007 | *G. hirsutum* | FN645929 |
|  |  | 2008 | *G. hirsutum* | GQ247893 |
|  |  | 2009 | *S. lycopersicum* | HM461862 |
|  |  | 2009 | *G. hirsutum* | HM461867 |
|  |  | 2009 | *G. hirsutum* | JF416947 |
|  |  | 2010 | *G. hirsutum* | JF510458 |
|  |  | 2010 | *G. hirsutum* | JN678805 |
| **CLCuKoV - Layyah** | Pakistan | 2011 | *G. hirsutum* | HF549182 |
| **CLCuKoV - Lucknow** | India | 2010 | *Cyamopsis tetragonoloba* | GU385879 |
| **CLCuKoV - Shadadpur** | Pakistan | 2005 | *G. hirsutum* | FN552001 |
| ***Papaya leaf curl virus* (PaLCuV)** | | | | |
|  | India | 1997 | *C. papaya* | Y15934 |
|  |  | 2006 | *S. lycopersicum* | DQ629102 |
|  |  | 2007 | *Jatropha gossypifolia* | EU727086 |
|  |  | 2007 | *Codiaeum variegatum* | JN831446 |
|  |  | 2007 | *Acalypha* sp. | FN645898 |
|  |  | 2007 | *C. tetragonoloba* | FN645915 |
|  |  | 2007 | *Acalypha* sp. | FN645926 |
|  |  | 2008 | *Crotalaria juncea* | GQ200446 |
|  |  | 2008 | *Raphanus sativus* | FJ593629 |
|  |  | 2008 | *C. variegatum* | JN817516 |
|  |  | 2009 | *Capsicum annuum* | JN663850 |
|  |  | 2010 | *Calotropis procera* | JQ407224 |
|  |  | 2010 | *Nicotiana glutinosa* | HM143914 |
|  |  | 2011 | *Amaranthus cruentus* | JN135233 |
|  |  | 2011 | *Aster alpinus* | JQ954859 |
|  |  | 2011 | *Glycine max* | JN807765 |
|  |  | 2011 | *Brassica rapa* | JX270684 |
|  |  | 2012 | *C. papaya* | KF307208 |
|  |  | 2012 | *C. variegatum* | AJ507777 |
|  | Pakistan | 2002 | *G. hirsutum* | AJ436992 |
|  |  | 2006 | *Croton glandulosus* | FN543112 |
|  |  | 2006 | *A. rosea* | FN678906 |
|  |  | 2007 | *Rhynchosia capitata* | FM955601 |
|  |  | 2012 | *G. hirsutum* | HG937524 |
| ***Okra enation leaf curl virus* (OELCuV)** | | | | |
|  | India | 2006 | *A. esculentus* | GU111996 |
|  |  | 2007 | *A. esculentus* | FN645922 |
|  |  | 2010 | *A. esculentus* | JX181786 |
|  |  | 2011 | *A. esculentus* | KC342220 |
|  |  | 2012 | *A. esculentus* | KC019308 |
|  |  | 2012 | *A. esculentus* | KP208672 |
|  |  | 2014 | *A. esculentus* | KT390308 |
|  |  | 2014 | *A. manihot* | KT390450 |
|  |  | 2014 | *H. cannabinus* | KT390454 |
|  |  | 2014 | *A. moschatus* | KT390459 |
|  |  | 2014 | *A. angulosus* | KT390458 |
|  | Iran | 2010 | *C. papaya* | KJ397528 |
|  | Pakistan | 2011 | *G. hirsutum* | HF567945 |
|  |  | 2012 | *A. esculentus* | HG518793 |
|  |  | 2012 | *A. esculentus* | HG938362 |
| ***Chickpea chlorotic dwarf virus* (CpCDV)** | | | | |
|  | Pakistan | 2010 | *G. hirsutum* | HE956706 |
|  |  | 2009 | *G. hirsutum* | HG313782 |
|  |  | 2010 | *G. hirsutum* | HE864164 |
|  |  | 2012 | *G. arboreum* | KT634301 |
|  |  | 2007 | *Cicer arietinum* | AM850136 |
|  |  | 2012 | *A. esculentus* | KT719391 |
|  |  | 2012 | *S. lycopersicum* | KP881605 |
|  |  | 2007 | *C. arietinum* | AM900416 |
|  |  | 2012 | *Cucumis sativus* | KT719388 |
|  |  | 2005 | *C. arietinum* | AM849097 |
|  |  | 2012 | *Lens culinaris* | KM377673 |
|  |  | 2011 | *X. strumarium* | HE610413 |
|  |  | 2010 | *C. arietinum* | KM377668 |
|  |  | 2010 | *L. culinaris* | KM377671 |
|  | India | 2011 | *C. arietinum* | JX183064 |
|  |  | 2011 | *C. arietinum* | JF831148 |
|  | Sudan | 2013 | *Vicia faba* | KM229785 |
|  |  | 2013 | *C. arietinum* | KM229774 |
|  |  | 2014 | *C. arietinum* | KM229778 |
|  |  | 2008 | *C. arietinum* | KM229768 |
|  |  | 2008 | *Pisum sativum* | KM229786 |
|  | Morocco | 2013 | *C. arietinum* | KM229788 |

| **CLCuD-associated bipartite begomoviruses** | **Country** | **Year of Collection / Submission** | **Host** | **Accession number** | |
| --- | --- | --- | --- | --- | --- |
|  |  |  |  | **DNA-A** | **DNA-B** |
| ***Tomato leaf curl New Delhi virus* (ToLCNDV)** | India | 1992 | *S. lycopersicum* | U15015 | U15017 |
|  |  | 1995 | *S. lycopersicum* | Y16421 | X89653 |
|  |  | 2002 | *S. tuberosum* | AY286316 | AY158080 |
|  |  | 2003 | *S. lycopersicum* | AY428769 | AY438563 |
|  |  | 2003 | *A. esculentus* | FJ561298 |  |
|  |  | 2004 | *A. esculentus* | GU112082 |  |
|  |  | 2005 | *S. lycopersicum* | DQ169056 | DQ169057 |
|  |  | 2005 | *L. cylindrica* | AY939926 | AY939924 |
|  |  | 2005 | *C. papaya* | DQ989325 |  |
|  |  | 2005 | *S. tuberosum* | EF043231 | EF043232 |
|  |  | 2005 | *A. esculentus* | GU112084 |  |
|  |  | 2005 | Pumpkin | AM286434 | AM286435 |
|  |  | 2005 | *G. hirsutum* | EF063145 |  |
|  |  | 2006 | *S. tuberosum* | AM850115 | FN356024 |
|  |  | 2006 | *A. esculentus* | EF035482 |  |
|  |  | 2006 | *S. lycopersicum* | EF068246 |  |
|  |  | 2006 | *A. esculentus* | GU112086 |  |
|  |  | 2006 | *A. esculentus* | GU112088 |  |
|  |  | 2006 | *C. annuum* | EU309045 |  |
|  |  | 2006 | Pumpkin | JN129254 |  |
|  |  | 2007 | *Lagenaria siceraria* | FN645905 |  |
|  |  | 2008 | *S. lycopersicum* | FJ468356 |  |
|  |  | 2008 | *S. lycopersicum* | HM345979 |  |
|  |  | 2008 | *S. lycopersicum* | HQ141673 | HQ141674 |
|  |  | 2009 | *C. annuum* | HM007113 |  |
|  |  | 2008 | *C. annuum* | HM007120 |  |
|  |  | 2009 | *S. lycopersicum* | HM159454 | HM159455 |
|  |  | 2009 | *S. lycopersicum* | GQ865546 |  |
|  |  | 2009 | *S. melongena* | HQ264185 |  |
|  |  | 2009 | *S. lycopersicum* | KF571461 |  |
|  |  | 2010 | *S. tuberosum* | KC874502 |  |
|  |  | 2011 | *S. tuberosum* | KC874507 |  |
|  |  | 2010 | *L. acutangula* | HM989845 | HM989846 |
|  |  | 2011 | *S. lycopersicum* | KF537780 |  |
|  |  | 2011 | *S. lycopersicum* | KF551576 |  |
|  |  | 2009 | *S. lycopersicum* | KF551582 |  |
|  |  | 2011 | *S. lycopersicum* | KF551589 |  |
|  |  | 2011 | *Benincasa hispida* | JN208136 |  |
|  |  | 2012 | *L. esculentum* | JX232220 |  |
|  |  | 2012 | *S. lycopersicum* | JX460805 |  |
|  |  | 2012 | *Papaver somniferum* | KC513822 |  |
|  |  | 2012 | *C. sativus* | KC545812 |  |
|  | Bangladesh | 2005 | *S. lycopersicum* | AJ875157 | AJ875158 |
|  |  | 2006 | *C. sativus* | EF450316 |  |
|  | Thailand | 1998 | Luffa | AF102276 |  |
|  |  | 2006 | *C. sativus* | AB330079 | AB330080 |
|  |  | 2006 | *L. siceraria* | AB368447 |  |
|  |  | 1996 | *C. sativus* | AB368448 |  |
|  |  | 2010 | *Sauropus androgynus* | JN809814 |  |
|  | Indonesia | 2008 | *C. sativus* | AB613825 | AB613826 |
|  | Taiwan | 2007 | *C. melo* | GU180095 | GU180096 |
|  | Pakistan | 1997 | *S. nigrum* | AJ620187 | AJ620188 |
|  |  | 2000 | *S. lycopersicum* | AF448059 | AY150304 |
|  |  | 2001 | *S. lycopersicum* | AF448058 | AY150305 |
|  |  | 2004 | *S. lycopersicum* | AM258977 | AM778833 |
|  |  | 2004 | *C. annuum* | DQ116880 | DQ116882 |
|  |  | 2004 | *S. nigrum* | DQ116885 |  |
|  |  | 2004 | *S. nigrum* | AM849548 | AM849547 |
|  |  | 2004 | *Momordica charantia* | AM491590 | AM709505 |
|  |  | 2005 | *L. cylindrica* | AM292302 |  |
|  |  | 2005 | *M. charantia* | AM747291 |  |
|  |  | 2006 | *L. acutangula* | EF620534 | EF620535 |
|  |  | 2006 | *Eclipta prostrata* | HQ658479 |  |
|  |  | 2009 | *S. lycopersicum* | FN435309 |  |
|  |  | 2012 | *Chenopodium album* | KC914896 |  |
|  |  | 2013 | *Rumex dentatus* | HG316125 |  |
|  |  | 2013 | *Convolvulus arvensis* | KC960492 |  |
|  |  | 2013 | *Parthenium hysterophorus* | KF002409 |  |
|  |  | 2013 | *G. hirsutum* | LN845962 |  |
|  |  | 2013 | *G. hirsutum* |  | LN845956 |
|  | Spain | 2012 | Zucchini | KF749224 | KF749227 |
|  |  | 2012 | Zucchini | KF749225 | KF749228 |
|  |  | 2013 | Zucchini | KF749223 | KF749226 |
|  |  | 2014 | *S. lycopersicum* | KM977733 | KM977734 |
|  | Tunisia | 2015 | Melon | KP979713 |  |
|  | Italy | 2015 | *Cucurbita pepo* | KU145141 | KU145142 |
| ***Tomato leaf curl virus* (ToLCV)** | India | 1999 | *S. lycopersicum* | AF413671 |  |
|  |  | 1999 | *S. lycopersicum* | AF449999 |  |
|  |  | 2001 | *S. lycopersicum* | AY190290 | AY190291 |
|  |  | 2006 | *S. lycopersicum* | DQ629101 |  |
|  |  | 2007 | *S. lycopersicum* | KP164862 |  |
|  |  | 2007 | *S. lycopersicum* | GQ994098 |  |
|  |  | 2008 | *S. lycopersicum* | EU573714 |  |
|  |  | 2008 | *S. lycopersicum* | HM625838 |  |
|  |  | 2009 | *S. lycopersicum* | KP178726 |  |
|  |  | 2012 | *P. vulgaris* | KF440686 |  |
|  |  | 2012 | *S. lycopersicum* | KF515618 |  |
|  |  | 2012 | *S. lycopersicum* | JX547015 |  |
|  |  | 2012 | *S. lycopersicum* | KF612318 |  |
|  |  | 2012 | *S. lycopersicum* | KR092195 |  |
|  |  | 2014 | *C. papaya* | KP725055 |  |
|  |  | 2014 | *Ocimum* | KP698314 |  |
|  |  | 2009 | *C. annuum* |  | KU196751* |
|  |  | 2010 | *C. annuum* |  | KP235538* |
|  | Nepal | 2000 | *S. lycopersicum* | AY234383 |  |
|  | Pakistan | 2005 | *X. strumarium* | FR819708 |  |
|  |  | 2013 | *G. hirsutum* | LN794214 | LN713269* |
|  |  | 2013 | *G. hirsutum* | LN794215 |  |
| ***Cotton yellow mosaic virus* (CYMV)** | Benin, Africa | 2014 | *G. raimondii* | KT454834 | KT454835 |
|  |  | 2014 | *G. raimondii* | KU683748 | KU683750 |

| **CLCuD-associated DNA-satellites** | | | | |
| --- | --- | --- | --- | --- |
| **Name of the DNA-satellite** | **Country** | **Year of Collection / Submission** | **Host** | **Accession number** |
| **Cotton leaf curl Multan betasatellite-Multan** | Pakistan | 1996 | *G. hirsutum* | AJ298903 |
|  |  | 1996 | *G. hirsutum* | AJ292769 |
|  |  | 1996 | *Hibiscus* sp. | AJ297908 |
|  |  | 2006 | *G. annumalum* | AM712315 |
|  |  | 2006 | *G. letifolium* | AM712319 |
|  |  | 2006 | *G. barbadense* | AM712321 |
|  |  | 2006 | *G. punctatum* | EU384579 |
|  |  | 2006 | *G. hirsutum* var. *punctatum* | EU384582 |
|  |  | 2006 | *G. annumalum* | AM712311 |
|  |  | 2008 | *N. benthamiana* | FJ861370 |
|  |  | 2009 | *Sonchus arvensis* | FN432359 |
|  |  | 1996 | *G. hirsutum* | AJ299443 |
|  |  | 2000 | *G. hirsutum* | AJ421678 |
|  |  | 2001 | *G. hirsutum* | AJ315700 |
|  | India | 1995 | *G. hirsutum* | AJ291601 |
|  |  | 1995 | *G. hirsutum* | AJ316037 |
|  |  | 2005 | *G. hirsutum* | DQ191161 |
|  | China | 2006 | *H. rosa-sinensis* | EF465536 |
|  |  | 2007 | *H. rosa-sinensis* | JN968574 |
|  |  | 2008 | *A. esculentus* | FJ770371 |
|  |  | 2008 | *H. rosa-sinensis* | GQ503176 |
|  |  | 2008 | *H. rosa-sinensis* | HQ455350 |
|  |  | 2009 | *H. rosa-sinensis* | HQ455358 |
|  |  | 2009 | *G. hirsutum* | GQ906588 |
|  |  | 2009 | *A. esculentus* | GU574207 |
|  |  | 2011 | *H. rosa-sinensis* | JQ943409 |
|  |  | 2012 | *H. rosa-sinensis* | JX286657 |
|  |  | 2011 | *H. rosa-sinensis* | JX286659 |
|  |  | 2011 | *A. esculentus* | JX286665 |
|  |  | 2011 | *Malvaviscus penduliflorus* | JQ424827 |
|  |  | 2011 | *H. rosa-sinensis* | JQ963626 |
|  |  | 2011 | *G. hirsutum* | JQ317604 |
|  |  | 2012 | *H. cannabinus* | KF444949 |
|  |  | 2012 | *H. rosa-sinensis* | JX861211 |
|  |  | 2012 | *H. rosa-sinensis* | JX914661 |
|  |  | 2012 | *G. hirsutum* | KC171655 |
|  |  | 2013 | *H. rosa-sinensis* | KF766952 |
|  |  | 2015 | *A. manihot* | KU254610 |
|  |  | 2015 | *A. manihot* | KX523679 |
|  |  | 2013 | *G. hirsutum* | KP762787 |
|  | Philippines | 2012 | *H. rosa-sinensis* | KF413617 |
| **Cotton leaf curl Multan betasatellites-Burewala** | Pakistan | 2002 | *G. hirsutum* | AM084379 |
|  |  | 2002 | *G. hirsutum* | AM084380 |
|  |  | 1999 | *S. lycopersicum* | AJ316035 |
|  |  | 2005 | *G. hirsutum* | FN554719 |
|  |  | 2006 | *G. hirsutum* | FJ607041 |
|  |  | 2006 | *G. darwinii* | EU384602 |
|  |  | 2006 | *G. barbadense* | AM712322 |
|  |  | 2007 | *G. hirsutum* | AM774307 |
|  |  | 2008 | *G. hirsutum* | HE601939 |
|  |  | 2006 | *G. hirsutum* | HE601941 |
|  |  | 2009 | *G. hirsutum* | HE601944 |
|  |  | 2006 | *G. hirsutum* (octaploid) | EU384589 |
|  |  | 2006 | *G. hirsutum* subsp. *latifolium* | EU384591 |
|  |  | 2006 | *G. davidsonii* | EU384592 |
|  |  | 2006 | *G. darwinii* | EU384605 |
|  |  | 2007 | *G. hirsutum* | AM774312 |
|  |  | 2011 | *G. hirsutum* | HF549188 |
|  |  | 2011 | *G. hirsutum* | HG000665 |
|  |  | 2008 | *N. benthamiana* | FJ861368 |
|  |  | 2009 | *G. hirsutum* | FR877537 |
|  |  | 2012 | *R. communis* | HE985228 |
|  |  | 2013 | *G. arboreum* | HG428700 |
|  |  | 2005 | *S. lycopersicum* | AM490309 |
|  | India | 2002 | *G. hirsutum* | AY083590 |
|  |  | 2002 | *G. hirsutum* | AY763123 |
|  |  | 2004 | *G. hirsutum* | AY744380 |
|  |  | 2004 | *G. hirsutum* | AY795604 |
|  |  | 2007 | *G. hirsutum* | EU862816 |
|  |  | 2008 | *G. hirsutum* | GQ370388 |
|  |  | 2002 | *G. hirsutum* | DQ364230 |
|  |  | 2003 | *G. hirsutum* | JF509752 |
|  |  | 2007 | *G. hirsutum* | FN658722 |
|  |  | 2006 | *G. hirsutum* | HM146308 |
|  |  | 2006 | *G. hirsutum* | HQ343201 |
|  |  | 2009 | *G. hirsutum* | HQ158008 |
|  |  | 2009 | *G. hirsutum* | JF416948 |
|  |  | 2008 | *G. hirsutum* | GQ259599 |
|  |  | 2008 | *G. hirsutum* | GQ369730 |
|  |  | 2008 | *G. hirsutum* | GQ249185 |
|  |  | 2008 | *H. cannabinus* | FJ159274 |
|  |  | 2006 | *H. cannabinus* | EU880231 |
|  |  | 2006 | *H. cannabinus* | EF614158 |
|  |  | 2006 | *H. cannabinus* | EF620564 |
|  |  | 2006 | *H. cannabinus* | EU825205 |
|  |  | 2005 | *H. sabdariffa* | DQ298137 |
|  |  | 2003 | *S. lycopersicum* | AY438561 |
|  |  | 2009 | *S. lycopersicum* | GQ374449 |
|  |  | 2008 | *C. tetragonoloba* | GQ370389 |
|  |  | 2008 | *C. juncea* | GQ369731 |
|  |  | 2010 | *C. papaya* | JX217745 |
|  |  | 2004 | *G. hirsutum* | JF502374 |
|  |  | 2009 | *G. hirsutum* | JF502377 |
|  |  | 2010 | *G. hirsutum* | JF502383 |
|  |  | 2005 | *G. hirsutum* | JF502375 |
|  |  | 2010 | *G. hirsutum* | HM461864 |
|  |  | 2010 | *G. hirsutum* | HQ257373 |
| **Cotton leaf curl Multan betasatellites-Shahdadpur** | Pakistan | 2005 | *G. hirsutum* | FN554723 |
|  |  | 2009 | *G. hirsutum* | HE601946 |
|  |  | 2006 | *G. hirsutum* | HE601940 |
|  |  | 2007 | *Spinacia oleracea* | HF568784 |
|  |  | 2006 | *G. punctatum* | EU384580 |
|  |  | 2008 | *G. hirsutum* | HE601938 |
|  |  | 2009 | *G. hirsutum* | HE602952 |
|  |  | 2006 | *G. hirsutum* | HE602948 |
|  | India | 2009 | *G. hirsutum* | HQ257372 |
| **Cotton leaf curl Gezira betasatellite** | Sudan | 1996 | *G. hirsutum* | AY669328 |
|  |  | 1996 | *G. hirsutum* | AY077797 |
|  |  | 1996 | Sida | AY077798 |
|  |  | 1996 | *A. esculentus* | AY077799 |
|  |  | 1996 | *A. esculentus* | AY044141 |
|  |  | 2000 | *G. hirsutum* | AY044140 |
|  |  | 2000 | *G. hirsutum* | AY044143 |
|  |  | 2005 | Datura | DQ644564 |
|  |  | 2007 | *A. esculentus* | FJ868829 |
|  | Burkina Faso | 2008 | *A. esculentus* | FN554579 |
|  | Niger | 2007 | *A. esculentus* | FJ469629 |
|  |  | 2007 | *A. esculentus* | EU727088 |
|  | Israel | 2011 | Whiteflies feeding on squash | KT099178 |
|  |  | 2011 | Whiteflies feeding on squash | KU095847 |
|  | Saudi Arabia | 2012 | *A. esculentus* | HG530542 |
|  | United Arab Emirates | 2013 | *A. esculentus* | KM279620 |
| **Cotton leaf curl Multan alphasatellite** | Pakistan | 1998 | *G. hirsutum* | AJ132344 |
|  |  | 1998 | *G. hirsutum* | AJ132345 |
|  |  | 1999 | *N. tabacum* | AJ512956 |
|  |  | 1997 | *S. lycopersicum* | AJ512955 |
|  |  | 2009 | *G. hirsutum* | FR873571 |
|  |  | 2009 | *G. hirsutum* | FR877532 |
|  |  | 2010 | *G. hirsutum* | HE978348 |
|  |  | 2010 | *G. hirsutum* | HF564600 |
|  |  | 2011 | *G. hirsutum* | HE979548 |
|  |  | 2011 | *G. hirsutum* | HE966424 |
|  |  | 2010 | *G. hirsutum* | KR816016 |
|  |  | 2013 | *G. hirsutum* | HG934391 |
|  | India | 2008 | *G. hirsutum* | GQ478667 |
|  |  | 2009 | *N. tabacum* | HQ180392 |
|  |  | 2009 | *S. lycopersicum* | GQ374450 |
|  |  | 2011 | Triticum aestivum | KC305093 |
|  |  | 2011 | *S. nigrum* | KJ028212 |
|  |  | 2012 | *G. hirsutum* | KF584012 |
| **Cotton leaf curl Burewala alphasatellite** | Pakistan | 2011 | *G. hirsutum* | HE965682 |
|  |  | 2011 | *G. hirsutum* | HE972277 |
|  |  | 2011 | *G. hirsutum* | HF567947 |
|  |  | 2011 | *G. hirsutum* | KR816012 |
|  |  | 2012 | *G. hirsutum* | HG530127 |
|  |  | 2012 | *G. hirsutum* | HG515072 |
|  |  | 2012 | *Ipomoea batatas* | HG515069 |
|  |  | 2012 | *G. hirsutum* | HG421035 |
|  |  | 2013 | *G. hirsutum* | KR816014 |
|  |  | 2012 | *G. hirsutum* | HG530123 |
|  |  | 2012 | *A. esculentus* | HG530126 |
|  |  | 2013 | *G. hirsutum* | LN845925 |
|  |  | 2013 | *G. hirsutum* | HG934393 |
|  | India | 2007 | *G. hirsutum* | FN658728 |
|  |  | 2009 | *A. esculentus* | HQ728354 |
|  |  | 2010 | *A. esculentus* | HM004548 |
|  |  | 2010 | *S. lycopersicum* | JX262389 |
|  |  | 2010 | *G. hirsutum* | GU992937 |
|  |  | 2010 | *G. hirsutum* | HQ316180 |
|  |  | 2014 | *G. hirsutum* | KM923999 |
| **Cotton leaf curl Shahdadpur alphasatellite** | Pakistan | 2005 | *G. hirsutum* | AM711115 |
| **Gossypium mustilinum symptomless alphasatellite** | Pakistan | 2006 | *G. gossypioides* | FJ218494 |
|  |  | 2006 | *G. latifolium* | FJ218495 |
|  |  | 2006 | *G. lobatum* | FJ218496 |
|  |  | 2006 | *G. mustelinum* | EU384662 |
| **Gossypium davidsonii symptomless alphasatellite** | Pakistan | 2006 | *G. mustelinum* | EU384655 |
| **Gossypium darwinii symptomless alphasatellite** | Pakistan | 2006 | *A. rosea* | FR772084 |
|  |  | 2006 | *G. darwinii* | EU384606 |
|  |  | 2006 | *G. davidsonii* | EU384623 |
|  |  | 2006 | *G. mustelinum* | EU384631 |
|  |  | 2006 | *G. hirsutum* (octaploid) | EU384644 |
|  |  | 2006 | *G. hirsutum* var. *punctatum* | EU384651 |
|  |  | 2006 | *G. tomentosum* | FJ218493 |
|  |  | 2009 | *X. strumaium* | HF547408 |
|  |  | 2010 | *G. hirsutum* | FR877533 |
|  |  | 2011 | *L. cylindrica* | HF567944 |
|  |  | 2011 | *G. hirsutum* | HE972276 |
|  |  | 2011 | *G. hirsutum* | HE965678 |
|  |  | 2012 | *G. hirsutum* | HG530128 |
|  | Oman | 2012 | *A. esculentus* | KF267445 |
|  | India | 2001 | *S. lycopersicum* | KF471038 |
|  |  | 2006 | *C. annuum* | KF471037 |
|  |  | 2007 | *G. hirsutum* | FN658729 |
|  |  | 2008 | *C. annuum* | KF471058 |
|  |  | 2008 | *C. annuum* | KF471040 |
|  |  | 2010 | *C. papaya* | JQ322970 |
|  |  | 2013 | *G. hirsutum* | KM103525 |
|  |  | 2013 | *G. hirsutum* | KM070824 |
|  |  | 2013 | *A. esculentus* | KT390426 |
|  |  | 2014 | *A. esculentus* | KJ843307 |
|  |  | 2014 | *G. hirsutum* | KT228322 |
| **Cotton leaf curl Gezira alphasatellite** | Cameroon | 2007 | *A. esculentus* | HE858192 |
|  | Burkina Faso | 2008 | *A. esculentus* | FN554581 |
|  | Sudan | 2010 | *A. rosea* | HM446369 |
|  |  | 2011 | *S. lycopersicum* | KC763631 |
|  | Israel | 2011 | Whiteflies feeding on squash | KT099176 |
|  | Saudi Arabia | 2012 | *A. esculentus* | HG530544 |

Common names of host plants mentioned in the table are as follows: *Abelmoschus esculentus* (Okra), *Alcea rosea* (Hollyhock), *Benincasa hispida* (Ash gourd), *Capsicum annuum* (Chilli), *Carica papaya* (Papaya), *Cicer arietinum* (Chickpea), *Codiaeum variegatum* (Croton), *Crotalaria juncea* (Sunn hemp), *Cucumis melo* var. *makuwa* cv. Silver Light (Oriental melon), *Cucumis sativus* (Cucumber), *Cyamopsis tetragonoloba* (Guar), *Glycine max* (Soybean), *Gossypium hirsutum* (Cotton), *Hibiscus cannabinus* (Kenaf), *Hibiscus sabdariffa* (Roselle), *Ipomoea batatas* (Sweet potato), *Lagenaria siceraria* (Bottle gourd), *Lens culinaris* (Lentil), *Luffa acutangula* (Vegetable gourd), *Luffa cylindrical* (Sponge Gourds), *Momordica charantia* (Bitter gourd), *Papaver somniferum* (Poppy), *Phaseolus vulgaris* (Common bean), *Pisum sativum* (Pea), *Raphanus sativus* (Radish), *Ricinus communis* (Castorbean), *Solanum lycopersicum* (Tomato), *Solanum melongena* (Eggplant), *Solanum tuberosum* (Potato), *Spinacia oleracea* (Spinach), Triticum aestivum (Wheat), *Vicia faba* (Broad bean), *Xanthium strumarium* (Cocklebur).

* CLCuAlV – Lobatum and – Multan strains (monopartite) were found associated with DNA-B. However, *Tomato leaf curl virus* was associated with DNA-B of ToLCNDV, while DNA-B of ToLCV was found to be associated with DNA-A of ToLCNDV.

All the begomovirus and CLCuMuB isolates were named and abbreviated according to Brown et al. (2015) and Akhtar et al. (2014), respectively.

**References:**

Akhtar, S., Tahir, M.N., Baloch, G.R., Javaid, S., Khan, A.Q., Amin, I., Briddon, R.W., and Mansoor, S. (2014). Regional Changes in the Sequence of Cotton Leaf Curl Multan Betasatellite. *Viruses* 6**,** 2186-2203. doi: 10.3390/v6052186

Brown, J.K., Zerbini, F.M., Navas-Castillo, J., Moriones, E., Ramos-Sobrinho, R., Silva, J.F., Fiallo-Olivé, E., Briddon, R.W., Hernández-Zepeda, C., Idris, A., Malathi, V.G., Martin, D.P., Rivera-Bustamante, R., Ueda, S., and Varsani, A. (2015). Revision of Begomovirus taxonomy based on pairwise sequence comparisons. *Arch. Virol.* 160**,** 1593-1619. doi: 10.1007/s00705-015-2398-y
